# The DREAM Initiative: study protocol for a randomized controlled trial testing an integrated electronic health record and community health worker intervention to promote weight loss among South Asian patients at risk for diabetes

**DOI:** 10.1186/s13063-019-3711-y

**Published:** 2019-11-21

**Authors:** Sahnah Lim, Laura C. Wyatt, Shinu Mammen, Jennifer M. Zanowiak, Sadia Mohaimin, Keith S. Goldfeld, Donna Shelley, Heather T. Gold, Nadia S. Islam

**Affiliations:** 1 Department of Population Health, NYU School of Medicine, 180 Madison Avenue, New York, NY 10016 USA; 2 Department of Population Health, NYU School of Medicine, 550 First Avenue, New York, NY 10016 USA

**Keywords:** South Asian, Community health workers, Electronic health records, Diabetes prevention, CBPR, Health disparities

## Abstract

**Background:**

Electronic health record (EHR)-based interventions that use registries and alerts can improve chronic disease care in primary care settings. Community health worker (CHW) interventions also have been shown to improve chronic disease outcomes, especially in minority communities. Despite their potential, these two approaches have not been tested together, including in small primary care practice (PCP) settings. This paper presents the protocol of Diabetes Research, Education, and Action for Minorities (DREAM) Initiative, a 5-year randomized controlled trial integrating both EHR and CHW approaches into a network of PCPs in New York City (NYC) in order to support weight loss efforts among South Asian patients at risk for diabetes.

**Methods/design:**

The DREAM Initiative was funded by the National Institute of Diabetes and Digestive and Kidney Diseases (National Institutes of Health). A total of 480 individuals at risk for type 2 diabetes will be enrolled into the intervention group, and an equal number will be included in a matched control group. The EHR intervention components include the provision of technical assistance to participating PCPs regarding prediabetes-related registry reports, alerts, and order sets. The CHW intervention components entail group education sessions on diabetes prevention, including weight loss and nutrition. A mixed-methods approach will be used to evaluate the feasibility, adoption, and impact (≥ 5% weight loss) of the integrated study components. Additionally, a cost effectiveness analysis will be conducted using outcomes, implementation costs, and healthcare claims data to determine the incremental cost per person achieving 5% weight loss.

**Discussion:**

This study will be the first to test the efficacy of an integrated EHR–CHW intervention within an underserved, minority population and in a practical setting via a network of small PCPs in NYC. The study’s implementation is enhanced through cross-sector partnerships, including the local health department, a healthcare payer, and EHR vendors. Through use of a software platform, the study will also systematically track and monitor CHW referrals to social service organizations. Study findings, including those resulting from cost-effectiveness analyses, will have important implications for translating similar strategies to other minority communities in sustainable ways.

**Trial registration:**

This study protocol has been approved and is made available on ClinicalTrials.gov by NCT 03188094 as of 15 June 2017.

## Background

South Asians in the US and New York City (NYC) are among the largest and fastest growing Asian subgroups [1–3]. Type 2 diabetes mellitus (DM) is a preventable chronic disease that disproportionately affects South Asians, both in their home countries and in the US [4–6]. A study conducted with community-based cohorts in two urban US cities reported a DM prevalence of 23% among South Asians compared to 6% in whites, 18% in African Americans, and 17% in Latinos [7]. Similarly, in NYC, both population-based self-report and clinically measured rates of pre-DM and DM are higher among South Asians compared to non-Hispanic whites [8–10]. Furthermore, Asian Americans often develop DM at a lower body mass index (BMI) compared to other racial/ethnic groups [11], informing the recommendation by the American Diabetes Association (ADA) to screen Asian Americans at a lower BMI threshold of 23 kg/m^2^ compared to 25 kg/m^2^ among the general population [12]. Considering that a significant portion of South Asians live in poverty, have limited English proficiency, and lack of access to culturally appropriate community resources [13], culturally tailored and effective interventions to prevent DM among South Asian Americans are sorely needed.

Results of the Indian Diabetes Prevention Program (DPP) and interventions conducted among South Asian immigrants in high-income countries have shown that modest weight loss through dietary changes and increased physical activity have significantly reduced the incidence of DM [14–16]. A few DPP adaptions (e.g., reduced number of sessions, cultural tailoring) for the South Asian community have demonstrated continued effectiveness in increasing weight loss, including studies that have utilized community health workers (CHWs) for program delivery [16–18]. With the advent of the patient-centered medical home model, primary care practices (PCP) increasingly aim to work in an integrated manner to coordinate care for a patient [19]. CHWs act as bridges between the community and healthcare system, and accordingly, there has been a growing evidence base for their integration into primary care settings to improve care for patients with chronic disease at low cost [20–22]. While previous work has demonstrated the efficacy of deploying CHWs to promote weight loss and diabetes prevention, there have been few studies on the effectiveness of integrating CHWs into primary care teams in order to facilitate diabetes prevention.

Data from electronic health records (EHRs) can improve primary and secondary prevention of DM by allowing for a more systematic identification and follow-up of patients at risk for DM [23]. Clinical decision support systems or alerts can increase provider adherence to screening and monitoring guidelines for DM prevention and management (e.g., hemoglobin A1c testing) [24–26]. The Asian American, Native Hawaiian, and Pacific Islander Diabetes Coalition launched the “Screen at 23” campaign to raise awareness of the need to screen Asian Americans for diabetes at lower BMI [27]. EHR-based tools may also be a promising approach [28] in specifically enhancing provider screening at lower BMI for our study population. Despite the tremendous potential that both CHW- and EHR-based interventions can have on improving health outcomes in minority communities, few studies have examined the impact of integrating the two interventions and how best to engage both physician and non-physician members of the healthcare team, including CHWs [29], in effectively integrating EHR-based interventions into care planning. Evaluations are needed for studies that are conducted in small primary care settings, as well as scalable and sustainable models that assess the cost, adoption, and maintenance process of integration into clinical practice settings.

This paper presents the protocol of a research study designed to understand the effectiveness of an integrated EHR and CHW intervention to promote weight loss among South Asian patients at risk for DM in 20 NYC PCPs. The research study is a 5-year randomized controlled trial (RCT) designed to test the feasibility, adoption, and impact of integrating CHW-led health coaching with EHR-based interventions to support weight loss for South Asian patients at risk of DM in primary care settings. It is hypothesized that individuals receiving care during the intervention will be more likely to lose ≥ 5% of their body weight compared to individuals receiving usual care.

## Methods/design

### Study team

The Diabetes Research, Education, and Action for Minorities (DREAM) Initiative is led by researchers from New York University (NYU) School of Medicine and is funded by the National Institute of Diabetes and Digestive and Kidney Diseases (National Institutes of Health). Researchers from NYU partnered with Healthfirst (HF), a not-for-profit managed care organization serving more than 35,000 South Asians in NYC. PCPs enrolled into this study are part of HF’s provider network [30] and serve large South Asians communities. The team also partners with the NYC Department of Health and Mental Hygiene Primary Care Information Project (PCIP) [31], a city-wide initiative seeking to improve population health through health information technology and data exchange. Specifically, PCIP supports the adoption and use of EHR among PCPs, working with over 1500 providers across the city. For this study, PCIP will provide participating PCPs with training and on-going technical assistance on the EHR component of the intervention. PCIP will also liaise directly with EHR vendors in the development, deployment, and testing of the pre-diabetes registries. In addition, the team engaged the All Medical Care Independent Physician’s Association (AMC IPA), which works with over 100 South Asian physicians in NYC. The AMC IPA will assist with the study’s PCP recruitment, distribution of educational material, and dissemination of study results throughout their physician network. Lastly, the study convened a community advisory board (CAB) comprised of South Asian community-based organizations who provide culturally tailored expertise in the development and implementation of the CHW intervention, including reviewing and adapting the CHW curriculum and participant materials [17, 32].

### Ethics and data sharing protocols

Final study protocol and procedures were reviewed and approved by the NYU School of Medicine Institutional Review Board on December 10, 2018. PCPs signed a Memorandum of Understanding (MOU) between the PCP and NYU outlining each party’s responsibilities: 1) components of the EHR intervention; 2) EHR training requirements; 3) EHR patient data extraction and confidentiality and storage procedures associated with the study; and 4) CHW intervention components. Written informed consent will be obtained from treatment group participants in the CHW arm. A Waiver of Authorization of consent was obtained for EHR extraction of de-identified clinical measures for control participants. This component of the intervention was registered via ClinicalTrials.gov by NCT03188094 as of June 15, 2017.

### Study design

The study is a two-arm RCT that analyzes the implementation and effectiveness of a multi-component intervention on weight loss promotion tailored for South Asians. The intervention consists of two components (EHR and CHW) within 20 HF network PCPs in NYC that serve a large proportion of South Asian patients. The 20 sites are roughly divided into three groups, in order for the intervention to be implemented in three successive waves (i.e., staggered) throughout the 3 years without overlap. Within each PCP site, individuals will be randomized to the CHW intervention or to usual care (control) group. The first year of the study was dedicated to recruitment of PCP sites, planning, and determination of specific components of the EHR and CHW interventions. During years 2 through 4, two intervention components will simultaneously be implemented within 20 PCP practice sites. For each PCP site, the EHR–CHW intervention is implemented in two rounds of 6-month phases, totaling one year of intervention. In year 5, the implementation process will be assessed and findings will be used to develop a set of best practices and toolkits for public health and healthcare agencies regarding integrated EHR–CHW strategies to promote weight loss.

**Fig. 1:**
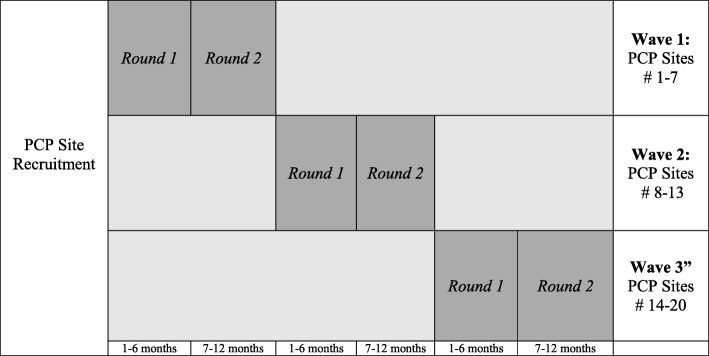
Staggered randomized controlled trial design for a weight loss intervention among South Asian patients at risk for diabetes

Figure 1 illustrates the staggered RCT design. There are three waves across a period of three years, and each PCP practice site has two rounds. Data related to the primary study outcomes are extracted from EHR systems on a bi-annual basis over a period of 18 months (baseline, 6-, 12-, and 18-month time points). The control group will receive usual care from their PCPs throughout study years 2 to 4. In study year 5, when all group sessions with treatment participants have ended, the control group participants will be offered the same group sessions as a point of service and not as a part of research.

#### First component: EHR intervention

Beginning in study year 2, the EHR intervention occurs simultaneously with the CHW intervention. Components of the EHR intervention were developed with input from project partners and participating PCP sites. These components are focused on: 1) generating routine patient registry reports that identify patients at risk for DM within each practice; and 2) developing and implementing medical alerts and order sets that are tailored to the South Asian patient population, including alerts to screening for BMI at ≥ 23 kg/m^2^. After consideration of the baseline workflow, staff capacity, and logical feasibility conducted through a mixed methods baseline assessment by PCIP, EHR intervention features are implemented at participating sites. Recommendations are then made by PCIP to create a revised practice workflow that ensures the proposed EHR intervention component is practical, realistic, and tailored toward each individual practice.

##### Patient registry

Within the EHR, there is a feature that allows providers to query and group patient information based on specific criteria, such as diagnoses of particular health conditions and demographic characteristics [33]. These features of the registry allow practices to plan and prioritize patient visits, identify potential care required, and measure overall practice performance. PCIP provides the clinical team with training on the following competencies: 1) understanding the functionality and potential impact of the registry; 2) generating registry reports for follow-up care; 3) identifying patients that have been lost to follow-up; incorporating registries into day-to-day office activities; and 4) monitoring the use, satisfaction, and impact of patient registry use over time. The focus of the project is on encouragement of the routine generation of registry reports in order to more systematically identify patients at risk for diabetes using HbA1c values between 5.7% and 6.4% at the last clinic visit; this will help providers to prioritize a follow-up visit from these patients.

##### Alerts

Alerts are built within the EHR in order to remind staff and providers to complete a particular action at the point of care [34]. Alerts can be patient-specific or may apply to any patient-satisfying specified criteria. PCIP provides the clinical team with training on how to understand the functionality and the potential impact of alerts, as well as how to utilize alerts for the prevention and management of DM. For our study, alerts are tailored to trigger for patients with an elevated BMI (≥ 23 kg/m^2^), in line with recommendations by the ADA for a lowered BMI cutoff point for DM in Asian American adults [35]. These alerts prompt PCPs to order an HbA1c test to further investigate DM risk.

##### Order sets

Order sets are linked to an alert and are standardized sets of evidence-based treatment guidelines [34]. In this project, we will create an order set that includes a combination of lab tests and counseling orders that are “pre-set” for patients at risk for DM. Within the counseling orders, evidence-based, culturally tailored, in-language educational materials are uploaded for distribution to patients in various South Asian languages (e.g., Hindi, Punjabi, Bengali, and Urdu).

In order to complement these components, participating PCP staff are trained on how to create customizable templates that will increase the efficiency and accuracy of the documentation of vital signs and other pertinent health data, as well as how to utilize automated appointment reminder texts and letters that can be sent to patients electronically [34]. PCIP will be providing initial training to providers and/or staff members that primarily focus on using the registry reports and documentation of screening for BMI at ≥ 23 kg/m^2^. PCIP will also review and emphasize the importance of collecting accurate weight measurements at every clinic visit, which will improve the quality of BMI data from the EHR (i.e., primary outcome). PCIP will conduct follow-up technical assistance visits at each site every 2 months for up to a period of one year after training in order to review initial training material and assist with troubleshooting the registry reports. Simultaneously, practices are encouraged to collaborate with PCIP in order to participate in EHR-based incentive programs, such as Meaningful Use (MU) and National Committee for Quality Assurance (NCQA) Patient-Centered Medical Home (PCMH) recognition [36, 37].

#### Practice recruitment enrollment

Working in concert with PCIP, CAB members, and the AMC IPA and building upon past successful collaborations [38], we have identified independent PCPs in Queens and Brooklyn that are part of the HF network and serve a significant number of South Asian patients (defined as practices with greater than 70% of patients identifying as South Asian). Practices were required to have an operating EHR, specifically eClinicalWorks (eCW) or MDLand, for at least 12 months prior to the time of enrollment [39, 40]. These sites were contacted by PCIP or the NYU study team staff by telephone in order to assess their eligibility in terms of their racial/ethnic makeup of patients and interest in the study. If interest was expressed by the representative over the phone, a site visit was scheduled with the study team. During the site visit, an MOU to participate in the intervention was signed by eligible and interested practices. The EHR component of the intervention is launched over a 7-day period following signing of the MOU.

#### Second component: CHW intervention

Following the launch of the EHR component of the intervention and prior to the launch of the CHW component at each site, NYU and PCIP staff will work in concert with PCP staff to identify the list of patients at risk for DM and eligible for the CHW intervention through the EHR registry report. Eligibility criteria include: 1) BMI ≥ 23 kg/m^2^; 2) HbA1c levels between 5.7% and 6.4% in the last 12 months; 3) an office visit with their provider in the last 12 months; 4) between the ages of 21 and 74 years; and 5) not pregnant at the time of screening.

Using the EHR registry, a list of eligible patients is generated at each participating PCP site. The data manager then finalizes this list for randomization by removing any past study participants, checking for family members using address and telephone numbers, and for some sites, removing individuals that are in a zip code for a different clinic site. Potential family members are randomized, and one individual is kept on the list. The data manager then performs a matched pair randomization within PCP cluster using the R software package ‘matching’, whereby a single individual is drawn from the list, and a control match is found from the remaining individuals based on age (SD = 0.5), gender (exact), and BMI (SD = 0.5). If a control match is not found, this individual is placed on the unmatched list. This matching process is repeated until everyone on the list has either been matched or placed on the unmatched list Individuals placed into the treatment group receive a letter from their physician inviting them to participate in the CHW intervention; CHWs follow up the letter with a telephone call. CHWs have been trained in core competencies, including cardiovascular disease, mental health, motivational interviewing, smoking cession, and other related topics [41].

Study data are collected and managed using REDCap [42, 43] electronic data capture tools hosted at NYU Langone Health. All treatment group individuals are contacted by the CHWs and encouraged to participate in the intervention. Participants complete an in-person or phone-based screening assessing baseline demographics and logistical information, such as preferred language and session availability, as well as confirming study eligibility, including pregnancy status. Treatment participants must sign a consent form in order to participate, which will be administered by the CHW before the first session.

The CHWs deliver a standardized curriculum on DM prevention that has been adapted from the DPP [44, 45]. The protocol consists of five monthly 60-min group health education sessions that provide the tools and strategies necessary to promote weight loss and prevent DM [17]. Sessions also include risk factors that are culturally relevant for South Asian populations (Table 1). All materials are culturally and linguistically adapted, and sessions employ adult learning techniques and group-based learning and activities.

**Table 1:** Community health worker (CHW) intervention curriculum

Session topic	Session overview	Tailored cultural components
Session 1: Diabetes overview	1. What is diabetes?	• Discussion of diabetes prevalence and increased risk of diabetes among South Asians
	2. Type I, type II, gestational diabetes, prediabetes	
	3. Risk factors	• Explanation of BMI and at-risk BMI in Asian communities
	4. Symptoms	• Dispelling common cultural misconceptions regarding diabetes
	5. Blood sugar (high/low)	
	6. Prevention of diabetes, diet, exercise, social support, and goal setting	
	7. Myths and facts about diabetes	
Session 2: Nutrition	1. Eating a balanced diet	• Photos of typical South Asian foods
	2. Healthy eating tips	• Identifying and limiting sweets high in fat and sugar and substituting sweets with fruits for dessert • Building a balanced plate following the Plate Method with traditional South Asian foods
	3. Overcoming barriers (e.g., eating out and in social situations)	• Managing expectations for eating outside the home • In language role play video on eating out in South Asian social setting
	4. Reading a nutrition label	• Using South Asian food labels
	5. Goal-setting for healthy eating	• Inclusion of the family cook within session/working with family cook to improve nutrition in the entire household
Session 3: Physical activity	1. Energy balance between foods and physical activity/calorie needs	
	2. Benefits of physical activity	• Discussion of the concept of “Saint-Soldier” in Sikhism, which promotes discipline in spiritual practice as well as in social responsibilities to family and community
	3. Forms of exercise	
	4. Preventing injuries/safety	
	5. Incorporating physical activity routines and goal-setting	
	6. Overcoming barriers	• Home-based exercise/activities for women • List of free, local community exercise classes
Session 4: Stress management	1. Effects of stress on physical and emotional health	
	2. Coping with different feelings	
	3. Stress management techniques	• Discussion around stigma associated with mental health problems such as depression • Herbal remedies for stress relief (e.g., fennel seed tea, ginger paste compress for the forehead)
	4. Family support/happy family relations	
Session 5: Diabetes complications	1. Diabetes overview	
	2. Heart disease and stroke	• Review of popular South Asian foods high in salt and fat and limiting these foods
	3. Managing diabetes	• In language role play on receiving a prediabetes/diabetes diagnosis
	4. Staying motivated and goal-setting	

Sessions are held in PCP offices and other community spaces, and multiple timeslots for each session are provided to accommodate varying schedules. Between sessions, CHWs follow up with participants by phone or in-person through a home or clinic visit; these visits are conducted bi-weekly, and CHWs engage in goal-setting activities related to weight loss or other health issues identified jointly by the patient and the CHW (e.g., physical activity or healthy eating).

Over the total intervention period (4 years), a total of 480 treatment group participants will be enrolled. Matched control group participants will never be contacted by the research team or participate in research activities. De-identified clinical measures will be collected for both treatment and control participants from EHR data for final analysis.

### Study recruitment

#### Data collection, measures, study outcomes, and analysis

##### Implementation and sustainability outcomes

The study will employ a mixed methods approach in order to assess challenges, barriers, and facilitators associated with the implementation and adoption process of the integrated EHR–CHW intervention, as well as to determine sustainability and scalability of the model to other payer organizations and health systems serving large immigrant populations. In order to capture data on utilization patterns of the alert and registry tools and integrated EHR–CHW initiatives, system files will be extracted on a quarterly basis that indicate date and time stamps and user logins for each time the tools were used. The frequency of these measures will be described and compared 12 months pre- and post-intervention. In order to assess physician attitudes around national pre-diabetes guidelines and satisfaction with the integrated intervention, baseline and 12-month follow-up surveys will be administered to all participating PCPs after completion of two rounds of the 6-month interventions at each site. These data will be tabulated and summarized descriptively in order to assess changes in attitudes from baseline to 12 months.

In order to explore barriers and facilitators to implementation and intervention adoption, qualitative in-depth interviews will be conducted with PCPs, healthcare payer representatives, and CHWs at baseline and 12-month follow-up. To assess fidelity of the CHW education sessions, fidelity checklists will be conducted during sessions, and CHWs will keep encounter logs of all interactions with study participants. Interview transcripts and encounter logs will be analyzed for common themes by two independent researchers using both deductive and inductive approaches. First, initial codes will be identified based on the study aims and relevant prior implementation research. The researchers will independently read the transcripts, identifying additional codes based on new themes or subthemes emerging from the data. Second, the researchers will meet with each other and the study research team to compare and discuss codes, resolve discrepancies, and iteratively develop a codebook with code names and meanings. Third, researchers will apply the codebook to the transcripts. Researchers will identify patterns, common themes, and connections among themes throughout the transcripts. Baseline interviews will be used to refine the intervention toolkit and to explore anticipated barriers and facilitators to implementing an integrated EHR–CHW intervention. Post-intervention interview and encounter log data will help to determine key barriers, facilitators, and lessons learned from staff and CHWs regarding the implementation of the intervention in PCP settings for DM prevention in an underserved population.

Lastly, the study will use Medicaid claims data to determine intervention and implementation costs. These costs include: 1) administering the program itself (e.g., phone calls, staff time associated with recruitment and CHW intervention); 2) patient/family time estimated from visits and claims data; and 3) healthcare utilization by program participants based on utilization data (e.g., hospitalizations, DM-related laboratory tests). Using average resource utilization and cost per patient (in the first 6 and 12 months of follow-up), we will compare resources incurred by the trial arms during the follow-up period at each time point. Our outcome will be the incremental cost per person achieving 5% weight loss. Cost data will be used to inform HF leadership of program costs for consideration of sustainability of CHW models by payer organizations.

##### Intervention outcomes and analysis

The primary outcome of interest is whether a patient reduces weight by 5% or more over 6 months of the CHW intervention. We will be comparing patients randomized to a CHW group with control group patients who receive usual care. Since the control group has been created based on pair-wise matching with intervention group participants by age, sex, and BMI, all analyses will adjust for these three covariates. Due to the binary outcome (the patient achieves at least 5% weight-loss or not), we will use logistic regression. To account for nesting in the data structure (patients nested within PCP), we will use a mixed effects generalized linear model with PCP-level random effects. This will address the possibility that the PCPs will have different levels of success in helping their patients (both the control group and intervention group) achieve the 5% threshold. Likewise, CHWs will likely have varying effects on patient weight loss. However, since seven CHWs will be delivering the intervention, we will treat the CHW as a fixed effect in the outcome model. The analysis will be conducted on an intent-to-treat basis; a patient randomized and enrolled to the CHW group will be analyzed as part of that group regardless of whether or not they participate in the program. The analysis will provide point estimates and 95% confidence intervals for the relative odds of achieving weight loss; the confidence interval estimates will reflect both the paired matching as well as the clustering in the study design.

The secondary outcomes of interest include changes in HbA1c, blood pressure, and BMI at 6 months, and sustained ≥ 5% weight loss at 12 months. For analyses of these secondary outcomes, similar mixed effects regression models (adjusting for matching variables) will be used to estimate the CHW effect. We will use the appropriate mixed effect model depending on whether or not the outcomes are continuous or binary. All analyses will be conducted using R (R Foundation for Statistical Computing, Vienna, Austria).

#### Power calculation

We conservatively estimate an 8% difference in effect size (i.e., ≥ 5% weight loss) when comparing intervention and control group participants. Accrual of 960 participants, evenly randomized to intervention or control groups, will provide greater than 80% power to detect this difference, using a two-sided, 0.05-level test (i.e., 48 participants—24 intervention and 24 control participants per PCP). These calculations assume a 15% loss to follow-up and the enrollment of 20 PCP sites.

## Discussion

Results of this weight loss promotion study among South Asian patients at risk for diabetes build upon the evidence base of the effectiveness of CHWs to provide culturally tailored education to minority populations and to fill an essential intermediary role between patients and providers [17, 46, 47]. Further, this study proposes a unique integration of EHR and community-based CHW interventions. While the efficacy of EHR and CHW interventions have been tested separately, evaluations testing the two components together are lacking. In the context of testing the EHR components of an intervention, studies occurring in the context of small PCP settings with limited resources are also important. Study findings can potentially provide scalable and sustainable models for other minority communities.

There are a few limitations to this study. The network of participating PCPs are selected based on geographic clusters in order to minimize CHW workload. Such a convenience sample may result in participation bias. Second, only individuals randomized to the treatment arm have the opportunity to choose participation. We have attempted to reduce this selection bias by removing the matched control each time the treatment individual declines to participate in the intervention. Third, the quality of the EHR data on important clinical measures (e.g., BMI) may differ across PCP sites; we have tried to minimize variation in quality by providing training and follow-up technical assistance to clinics on weight measurement. Finally, there is a possibility of contamination, as the control group participants might interact with intervention participants or the EHR intervention may positively influence the provider’s care of all patients in the clinic, including control group participants. In order to reduce this bias, we have modified our final randomization lists to include only one potential family member, based on address and phone number, in the randomization process.

Despite these limitations, this study is the first to test the efficacy of an integrated EHR–CHW intervention among an underserved population and in a practical, real world setting. We leverage cross-sector partnerships to enhance the study’s implementation and outcomes. Our study findings will have implications for translating similar strategies to other minority communities, including Asian, African American, and Hispanic Americans. Lastly, study results on cost-effectiveness will inform sustainability efforts for various stakeholders, including healthcare payers.

## Trial status

Protocol version: Protocol dated 1/15/2019.

Trial registration: ClinicalTrials.gov.

Registration number: NCT 03188094.

Date of trial registration: June 15, 2017.

Was this trial prospectively registered? Yes.

Date recruitment began: December 2018. Recruitment is ongoing.

Anticipated end date: April 2022.

## Data Availability

The data and materials generated during this study will be available from the corresponding author on request.

## Abbreviations

ADA: American Diabetes Association
AMC IPA: All Medical Care Independent Physician’s Association
BMI: Body mass index
CAB: Community Advisory Board
CHW: Community health worker
DM: Diabetes mellitus
DPP: Diabetes Prevention Program
DREAM: Diabetes Research, Education, and Action for Minorities
eCW: eClinicalWorks
EHR: Electronic Health Record
HF: Healthfirst
MOU: Memorandum of Understanding
MU: Meaningful Use
NCQA: National Committee for Quality Assurance
NYC: New York City
NYU: New York University
PCIP: Primary Care Information Project
PCMH: Patient-Centered Medical Home
PCP: Primary care practice
RCT: Randomized controlled trial
